# Targeted Analysis Reveals an Important Role of *JAK-STAT-SOCS* Genes for Milk Production Traits in Australian Dairy Cattle

**DOI:** 10.3389/fgene.2015.00342

**Published:** 2015-12-15

**Authors:** Sondur J. Arun, Peter C. Thomson, Paul A. Sheehy, Mehar S. Khatkar, Herman W. Raadsma, Peter Williamson

**Affiliations:** Faculty of Veterinary Science, University of Sydney, NSWSydney, Australia

**Keywords:** *JAK-STAT* pathway genes, *SOCS* family genes, qRT-PCR, association mapping, dairy traits

## Abstract

The Janus kinase and signal transducer and activator of transcription *(JAK-STAT)* pathway genes along with suppressors of cytokine signalling (*SOCS)* family genes play a crucial role in controlling cytokine signals in the mammary gland and thus mammary gland development. Mammary gene expression studies showed differential expression patterns for all the *JAK-STAT* pathway genes. Gene expression studies using qRT-PCR revealed differential expression of *SOCS*2, *SOCS*4, and *SOCS*5 genes across the lactation cycle in dairy cows. Using genotypes from 1,546 Australian Holstein-Friesian bulls, a statistical model for an association analysis based on SNPs within 500 kb of *JAK-STAT* pathway genes, and *SOCS* genes alone was constructed. The analysis suggested that these genes and pathways make a significant contribution to the Australian milk production traits. There were 24 SNPs close to *SOCS*1, *SOCS*3, *SOCS*5, *SOCS*7, and *CISH* genes that were significantly associated with Australian Profit Ranking (APR), Australian Selection Index (ASI), and protein yield (PY). This study supports the view that there may be some merit in choosing SNPs around functionally relevant genes for the selection and genetic improvement schemes for dairy production traits.

## Introduction

Hormones and cytokines play an essential role in the growth and differentiation of the mammary gland. This is reflected in differential expression of genes during mammary gland development, and across different stages of lactation (Alluwaimi and Cullor, 2002). The cellular responses to cytokine signals are controlled by complex networks of intracellular signaling pathways which, despite the diversity of cytokines and growth factors, are highly conserved. The Janus kinase and signal transducer and activator of transcription (*JAK-STAT*) pathways are particularly important during lactation. Four different JAK kinases, namely JAK1, JAK2, JAK3, and TYK2, and seven different STAT members, namely STAT1, STAT2, STAT3, STAT4, STAT5a, STAT5b, and STAT6, have been described (Imada and Leonard, 2000). Various members of these gene families are key regulators of alveolar proliferation and differentiation in the mammary gland. Gene deletion analysis in mice confirms the mandatory requirement of the *JAK-STAT* signaling pathway in mammary gland development and lactation (Hennighausen et al., 1997; Yamaji et al., 2013).

A key regulatory feature of this pathway is a family of genes that encode a group of negative inhibitors named suppressors of cytokine signalling (*SOCS*; Linossi et al., 2013; Linossi and Nicholson, 2015). The *SOCS* family is required for the attenuation of cytokine signals in mammary epithelial cells, and acts to limit proliferation through a negative feedback mechanism. The *SOCS* family is comprised of eight members named *SOCS*1 to *SOCS*7 and *CISH* (cytokine-inducible SH2), and are characterized by a central Src-family homology 2 (SH2) domain and C-terminal *SOCS* box (Jegalian and Wu, 2002). *SOCS*1 and *SOCS*2 are required for the attenuation of the prolactin receptor signaling pathway during pregnancy and lactogenesis (Ramanathan et al., 2008; Riley et al., 2010; Wei et al., 2013). *SOCS*3 has been credited with control of the regulation of involution of mammary tissue through programmed cell death and tissue remodeling, initiated after the termination of lactation (Sutherland et al., 2007). Differential expression of *SOCS*3 relative to controls correlates with failed lactation in prolactin receptor knockout and galanin knockout mice (Naylor et al., 2005).

The present study examined gene expression of five *SOCS* family members across the lactation cycle in dairy cows. Additionally, we report the design and validation of a dairy trait association model using a targeted *JAK-STAT* pathway candidate gene approach, primarily focusing on the *SOCS* family genes.

## Materials and Methods

### Animal Selection and Collection of Mammary Tissue

Five multiparous Holstein-Friesian cows entering their third or fourth lactation were evaluated for milk production and had an Australian selection index (ASI) value in the top 25% of the Australian herd, and a previous lactation production range of 5,300–8,800 L per lactation. Mammary tissue biopsy samples were collected as described (Sheehy et al., 2009) serially from the five animals at three different time points either 5 days following termination of milking from the previous lactation (involution sample), approximately 20 days (8–23 days, average 17.8 days) prior to calving (pregnancy sample), and approximately 30 days (30–35 days, average 33.2 days) following calving (lactation sample). At the time of tissue sample collection, each animal was evaluated for body condition score. No pathogens were observed in any milk samples (as determined by culture) at the time of biopsy and the absence of negative energy balance was determined by blood metabolite analysis. All work with animals was conducted in accordance with the guidelines of the Animal Research Act, NSW, Australia, and was approved by the Animal Ethics Committee of the University of Sydney.

### RNA Extraction and Expression Studies using qRT-PCR

Approximately 100 mg of mammary tissue from each biopsy was treated with 1 ml of Tri-reagent (Sigma–Aldrich Pty. Ltd., NSW, Australia) and the RNA extracted was quantified by spectrophotometry. Approximately 100 μg of purified RNA was further purified by RNeasy column including an on-column DNase1 treatment (QIAgen, Doncaster, VIC, Australia). Single stranded cDNA was synthesized from 2 μg of RNA according to the manufacturer’s protocol using SuperScript III (Invitrogen Aust. Pty, Melbourne, VIC, Australia). Oligonucleotide PCR primers were manufactured by a commercial manufacturer (Sigma–Aldrich Pty. Ltd., NSW, Australia). The details of the primers for the five members of *SOCS* genes and the house keeping control gene used for relative quantification are summarized in **Table 1**. PCR was performed in the presence of Sybr Green and monitored for real time analyses using a Rotor-gene 6000 instrument (QIAgen, Doncaster, VIC, Australia), over 35 cycles of 95°C, 30 s, 60°C 30 s, 72°C 1 min. Each gene was analyzed for 15 different samples (*n* = 5 in each stage of lactation cycle) in triplicate and relative quantification was measured against a standard housekeeping gene, namely Large Ribosomal Protein (RPLP0). Statistical analysis to compare expression levels across the three stages of lactation was undertaken using a balanced ANOVA, incorporating cow as a blocking term, with statistical significance between the comparisons determined by a protected Fisher least significance difference (GenStat; VSN International Ltd., Hemel Hempstead, UK).

**Table 1:** Details of primers used for qRT-PCR.

Gene Name	Gene bank #	Primer Sequence 5′–3′		Expected product length
*SOCS1*	XM_864316.2	Forward	CTGGTTGTCGTAGCAGCTTAAC	134 bp
		Reverse	AATAAAGCCAGAGACCCTCC	
*SOCS2*	NM_177523.2	Forward	GGGACTGCCTTTACCAACAA	395 bp
		Reverse	GTGCTGGGACCTTTCACCTA	
*SOCS3*	NM_174466.2	Forward	CCCCCAGGAGAGCCTATTAC	153 bp
		Reverse	GGCAGCTGGGTGACTTTCT	
*SOCS4*	NM_001076218.1	Forward	AGCCAAGAAAGGAAGCACAG	163 bp
		Reverse	GATGGAAGCCCTGAAGAATG	
*SOCS5*	NM_001046182.1	Forward	ATGGGGACAGTTGTGCAGTT	159 bp
		Reverse	TCAATCTGCGTGTGGACTTT	
*RPLP0*	NM_001012682.1	Forward	CACTGTCCACGCCATCACT	226 bp
		Reverse	CCTGCTTCACCACCTTCTTG	

### Selection of SNPs

Genotyping data from 1,546 Australian Holstein-Friesian bulls was available for analysis as previously described (Khatkar et al., 2007, 2008). The SNPs within 500 kb of all *SOCS* genes, and genes associated with the *JAK-STAT* pathway, were collated for analysis. Each set of SNPs was analyzed for association against the range of dairy traits, as quantified by the Australian Breeding Value (ABV) for these milk production traits, obtained from the Australian Dairy Herd Improvement Scheme^1^. The traits included were the Australian Profit Ranking (APR), ASI, protein yield (PY), protein percentage (PP), milk yield (MY), fat yield (FY), and fat percentage (FP), and were analyzed for all 1,546 bulls.

The set of SNPs in the gene region was considered as a genotype, and this was conducted by concatenating the number of copies of the minor allele (e.g., “0-2-1” for a set with three SNPs). Then the associations between the genes (as assessed by constructed genotypes) and the traits were evaluated through fitting general linear models in GenStat, with the significance of each gene being assessed with an *F*-test. A backward elimination procedure was used to arrive at a minimum number of significant genes in the model, starting from a full model where all eight *SOCS* family genes, and 21 genes from *JAK-STAT* pathway genes were included. Genes were removed from the model using a threshold of *P* > 0.05. At the conclusion of the elimination procedure, the *P*-values, the contribution to total variation (*R*^2^) and the adjusted contribution (adjusted *R*^2^) for all genes was obtained. The procedure was repeated using *SOCS* genes only.

A simulation model was then developed to validate the results obtained for *JAK-STAT*-*SOCS* genes. The loci used for this model were selected randomly from the dataset. Following the same procedure, SNPs were selected within a distance of 500 kb of each locus. Subsequently, each set of SNPs was analyzed and the least significant were removed. One thousand permutations of this procedure were performed with SNPs selected at random from different locations each time. For comparison with results from the *JAK-STAT*-*SOCS* results, the distributions of *P*-values, the contribution to total trait variation and the adjusted variation were plotted for the 1000 permutations. This permutation analysis was conducted with a user-written procedure in R.^2^

## Results

### SOCS Gene Expression

**FIGURE 1:**
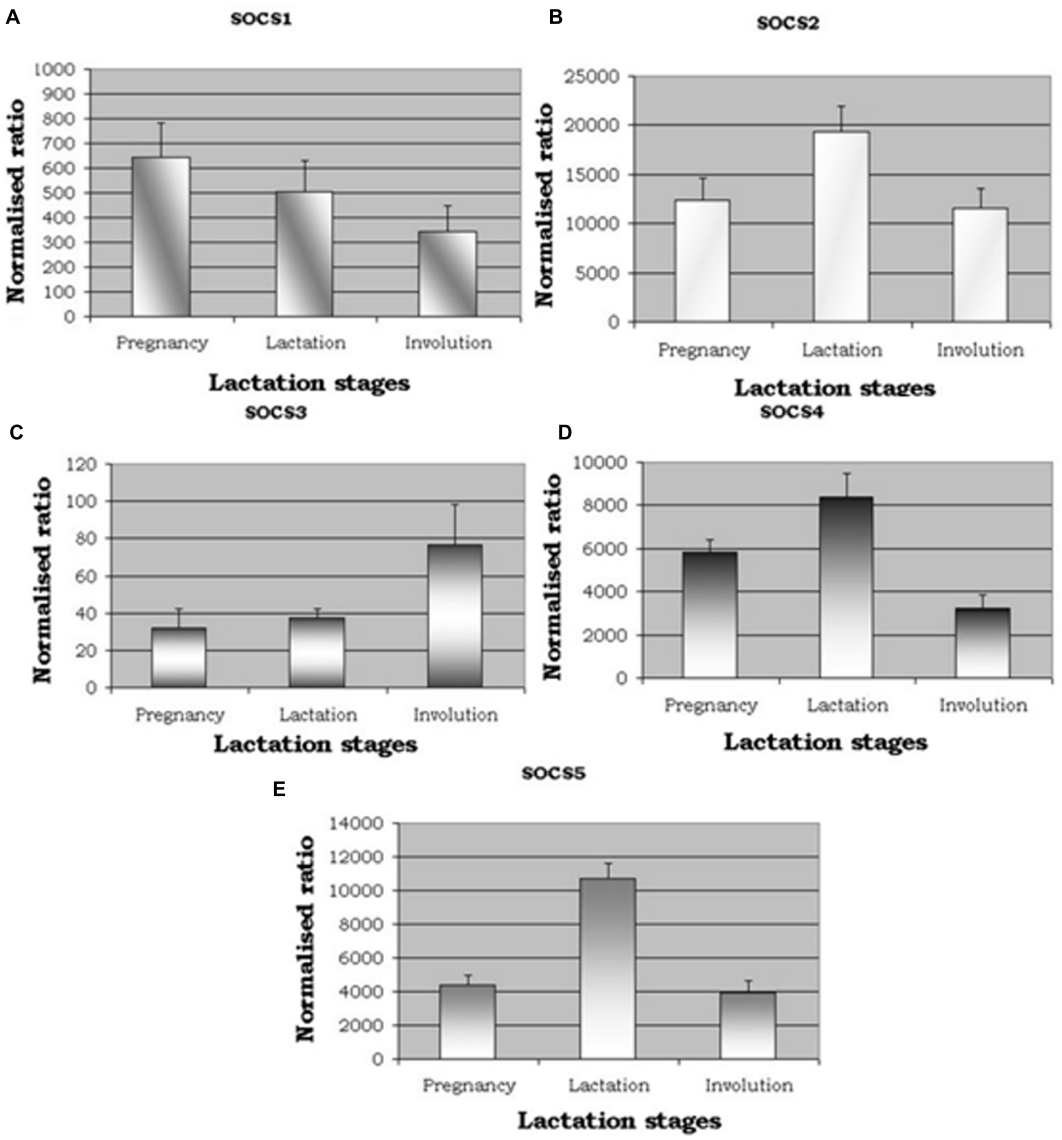
**qRT-PCR expression for suppressors of cytokine signalling (*SOCS)*. (A–E)** show expression of *SOCS*1, *SOCS*2, *SOCS*3, *SOCS*4, and *SOCS*5. Normalized ratios showing altered expression for five members of *SOCS* genes across the three distinct lactation stages, namely pregnancy, lactation, and involution. *P*-values were significant between lactation stages for [*SOCS2, P* = 0.057 (suggestive); *SOCS4, P* = 0.007); *SOCS5, P* < 0.001].

qRT-PCR was used to analyze *SOCS*1–*SOCS*5 gene expression across the three stages of the lactation cycle. When considering the relative levels of gene expression compared to the control gene (RPLPO), i.e., the ‘normalized ratio,’ expression of *SOCS*2 was notably greater, followed by *SOCS*5 (**Figure 1**). Overall levels of *SOCS*1, *SOCS*2, and *SOCS*3 were much lower. The patterns of expression were either, increased in lactation compared to the other stages (*SOCS*2, *SOCS*4, *SOCS*5), decreasing from pregnancy to involution (*SOCS*1), or increasing from pregnancy to involution (*SOCS*3). Statistically, there was no difference seen between animals (*n* = 5) at each stage (all *P* > 0.05), but a difference was seen between lactation stages for *SOCS*2 (*P* = 0.057, suggestive), *SOCS*4 (*P* = 0.007) and *SOCS*5 (*P* < 0.0001) based on ANOVAs. Further analysis based on Fisher’s protected LSD revealed that *SOCS*2, *SOCS*4, and *SOCS*5 were differentially expressed during lactation compared to the other two stages, but not when levels during pregnancy were compared to expression levels during involution.

### Analysis of Genotyping Data

A total of 98 SNPs were identified from the neighborhoods of eight *SOCS* genes and 21 *JAK-STAT* pathway genes, and were then collated into a group of SNPs representing all 29 genes. A preliminary analysis of SNPs around each gene revealed a significant association with each of the seven dairy traits tested. Subsequently, a general linear model was fitted to calculate the total variation explained by all SNPs across all the genes. After removing less significant predictors using a backward elimination procedure, the final gene-SNP combinations found to be significantly associated with each dairy trait were collated (**Tables 2** and **3**).

**Table 2:** Janus kinase and signal transducer and activator of transcription **(***JAK-STAT)* pathway gene combinations associated with dairy traits.

Dairy trait	Gene combinations	*P*-value range^∗^
APR	*SOCS*1 + *SOCS*3 + *CISH* + *IL*6*R* + *PRLR* + *STAT*2 + *IL*2	<0.001–0.018
ASI	*SOCS*3 + *JAK*1 + *IL*10 + *IL*3 + *IL*2	<0.001–0.001
Protein yield	*SOCS*5 + *CISH* + *STAT*2 + *IL*3 + *IL*2	<0.001–0.004
Protein percentage	*SOCS*3 + *SOCS*4 + *SOCS*6 + *SOCS*7 + *CISH* + *PRLR* + *STAT*2 + *IL*10 + *IL*3	<0.001–0.046
Fat yield	*SOCS*1 + *PRLR* + *IL*4*R* + *STAT*1 + *IL*3	<0.001–0.004
Fat percentage	*SOCS*6 + *STAT*6 + *PRLR* + *STAT*2 + *EPO* + *IL*10	<0.001–0.036
Milk yield	*SOCS*3 + *JAK*1 + *IL*10 + *IL*3 + *IL*2 + *JAK*2	<0.001–0.032

*^∗^The range of *P*-values correspond to the results of 1000 permutations.*

**Table 3:** Suppressors of cytokine signalling gene SNP combinations associated with dairy traits.

Dairy trait	Gene combinations	*P*-value range^∗^
APR	*SOCS*1 + *SOCS*3 + *SOCS*5 + *SOCS*7 + *CISH*	<0.001–0.005
ASI	*SOCS*1 + *SOCS*3 + *SOCS*5 + *SOCS*7 + *CISH*	<0.001–0.011
Protein yield	*SOCS*1 + *SOCS*3 + *SOCS*5 + *SOCS*7 + *CISH*	<0.001–0.020
Protein percentage	*SOCS*3 + *SOCS*4 + *SOCS*6 + *SOCS*7 + *CISH*	<0.001–0.010
Fat yield	*SOCS1* + *SOCS3* + *CISH*	<0.001–0.003
Fat percentage	*SOCS*1 + *SOCS*3 + *SOCS*6	<0.001–0.001
Milk yield	*SOCS*1 + *SOCS*3 + *SOCS*6 + *SOCS*7 + *CISH*	<0.001–0.049

*^∗^The range of *P*-values correspond to the results of 1000 permutations.*

When all genes from *JAK-STAT* pathways, including *SOCS* genes, were considered, the optimized set accounted for 28.1% of the total adjusted variation for APR. The range for the other six traits was from 20.8% for ASI down to 10.3% for FP (**Table 4**).

**Table 4:** Adjusted total variation explained by SNP combinations.

Dairy trait	*JAK-STAT* Pathway	*SOCS* only
APR	28.1%	23.7%
ASI	20.8%	18.5%
Protein yield	23.2%	20.8%
Milk yield	18.8%	14.4%
Protein percentage	19.9%	10.5%
Fat yield	10.9%	7.3%
Fat percentage	10.3%	6.2%

A model was also developed based only on SNPs around eight members of the *SOCS* family genes. Here, there was a more conservative removal of SNPs during elimination. The final combinations accounted for 23.7% of the adjusted total variation for APR (**Table 4**). The range for the remaining traits was from 20.8% for PY to 6.2% for FP. Notably, the total adjusted variation explained for PY was equivalent for both the *JAK-STAT* pathway genes together and the *SOCS* genes alone. The effects of various SNP combinations around the eight members of *SOCS* genes for each dairy trait were estimated. In general, there was a greater number of combinations with a positive rather than a negative effect, especially for PY, FY, FP, and MY. In contrast, there was a greater number of SNP combinations with a negative rather than positive effect on PP.

### Simulation Model

**FIGURE 2:**
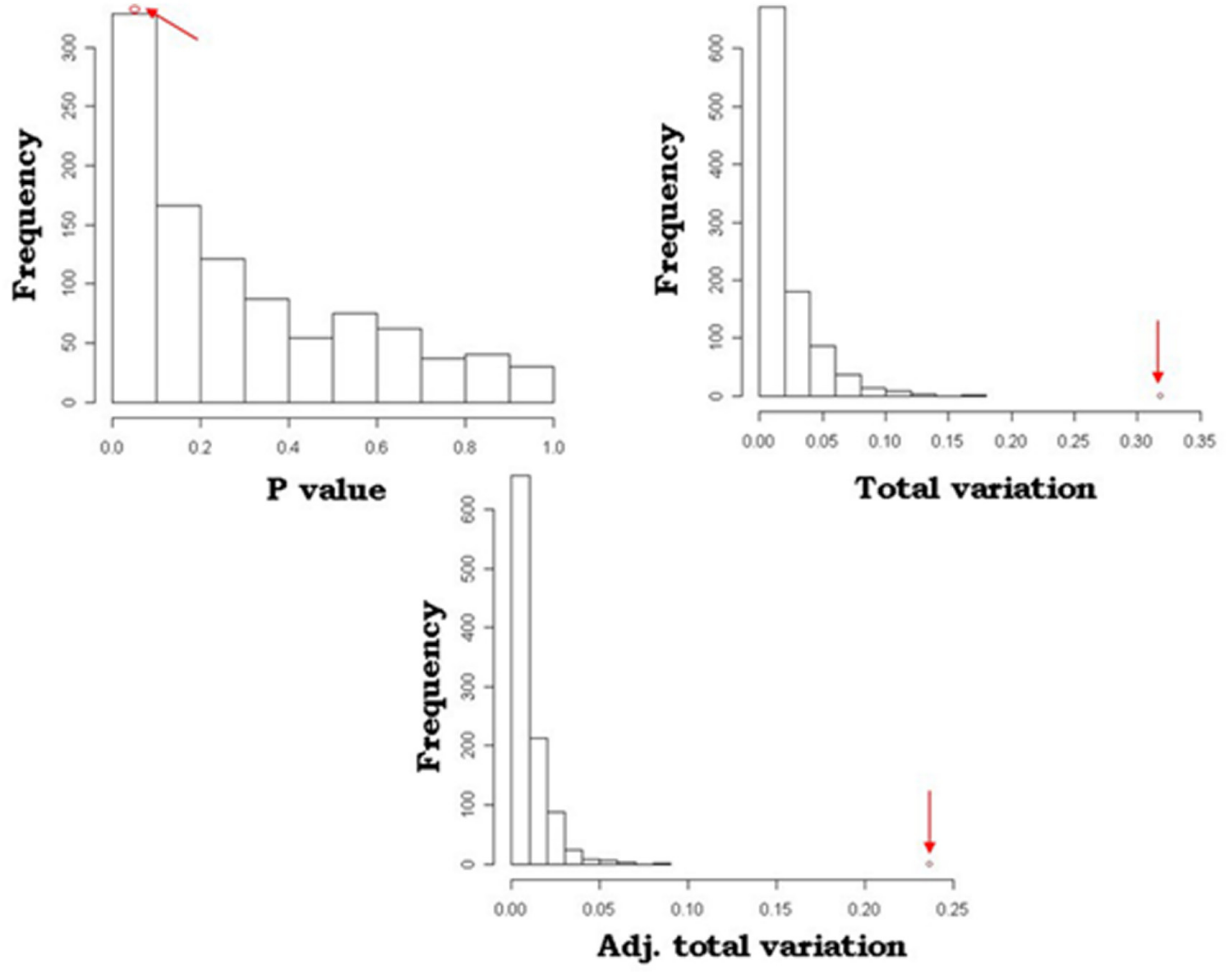
**Comparison of *SOCS* model and simulation outcomes for Australian Profit Ranking (APR).** Distribution of *P*-values, total variation and adjusted total variation for the 1000 permutation calculations in the simulation model. The arrows indicate the values identified in the *SOCS* association model.

A simulation model was developed to mimic and validate the results of the *SOCS* association model. With 1000 permutations for each trait, this resulted in three or fewer significantly associated loci for any of the traits, i.e., the probability of randomly identifying these loci was 0.003. An example of the comparative analysis between the simulation and *SOCS* model for APR is depicted in **Figure 2**. This shows the results of the observed data, as indicated by the red arrows, in comparison to the distribution of random SNP-gene associations. For all measures, the observed values are at the extreme end of the distributions, confirming the true statistical significance of these associations.

## Discussion

Genes that are functionally relevant to any biological process are often differentially regulated, both temporally and between physiological states. Members of the *JAK-STAT* pathways, and *SOCS* genes in particular as negative regulators, play an important role in mammary gland growth and development as mediators of hormone and cytokine signals (Chen et al., 2000; Alluwaimi and Cullor, 2002; Jegalian and Wu, 2002). In the present study, gene expression analysis identified that *SOCS*2, *SOCS*4, and *SOCS*5 genes were differentially expressed in mammary tissue across the lactation cycle in dairy cows. Focusing on the *JAK-STAT* pathways, a model was developed for *JAK-STAT* pathway genes, and separately for *SOCS* genes, to test association with dairy traits. The model revealed significant associations with genetic variants linked to some *JAK-STAT* pathway members, and specific members of the *SOCS* family. Further, the simulation study by randomly selecting genes as trait predictors indicated that the genes detected were indeed strongly associated, given the extremes they had on the distributions on all the metrics (**Figure 2**).

Gene expression analysis presented here showed that there is a relatively high level of expression of *SOCS*2, *SOCS*4, and *SOCS*5 in lactation when compared to the other two stages, whereas by comparison, *SOCS*1 and *SOCS*3 expression levels were 10-fold lower in relative terms, and showed a pattern in which expression during lactation was intermediate between that seen in the other two stages. The patterns of *SOCS*1 and *SOCS*2 expression are consistent with those measured in a study of photoperiod effects on dairy cattle (Wall et al., 2005; Dahl, 2008). In that study, the cows showed the highest expression of *SOCS*1 during pregnancy and the lowest expression during involution, and *SOCS*2 expression was highest during lactation. The pattern of *SOCS*3 expression was different between the studies, but in our study there was a very low level of *SOCS*3 expression and more variable. However, prolactin levels are raised during peak lactation and lower levels of *SOCS*3 expression may be expected, as noted in another study by those same authors (Wall et al., 2006). Other research has suggested a primary role of *SOCS*3 in apoptosis and tissue remodeling during involution (Sutherland et al., 2007), and *SOCS*3 activity may also reflect leukocyte infiltration and activation during this stage.

Expression of *SOCS* mRNA levels in the liver of dairy cattle are also elevated during lactation, consistent with insulin effects (Winkelman et al., 2008; Cummins et al., 2012). A similar pattern was seen in the present study in the mammary tissues of cows during lactation when compared to pregnancy and involution. Although *SOCS*3 expression was not influenced by genotype (Cummins et al., 2012), variation in *SOCS*2 expression could potentially affect milk production traits.

There have been several studies that have used SNP selection methods based on co-expression or other functional parameters to assess genetic contributions to production traits in cattle [see e.g., (Reverter and Chan, 2008; Moser et al., 2009, 2010; Raven et al., 2014; Widmann et al., 2015)]. Here, eight candidate genes from the *SOCS* family and 21 regulatory *JAK-STAT* pathway candidates were selected for analysis based on the generic function of these genes and their potential for involvement in biological processes relevant to the lactation cycle. *JAK-STAT* pathways act directly in providing intracellular signals that co-ordinate gene transcription in response to a wide range of cytokines and hormones. Some of these pathways have specific roles in mammary development and the lactation cycle, while others provide signals that are common in cellular responses. The strategy employed here was to capture family members and regulators without specifically selecting genes from within the family based on *a priori* knowledge. Some emphasis was placed on SOCS genes on the basis that negative regulators of intracellular pathways have the potential to have high impact. That is, in a network of interactions, negative regulators of key pathways are often represented as substantial nodes. Using this strategy, significant associations were identified, initially for SNP combinations from both the *JAK-STAT* members and *SOCS* family genes together, and subsequently for *SOCS* family genes alone. Interestingly, the genes neighboring the SNP combinations that were selected from the backward elimination procedure did not align with those that may have been expected, either from the *SOCS* expression data, or known involvement in lactation studies. Specifically, *SOCS*2 and STAT5 genes were not represented in the model. This is in contrast to the model developed by Raven et al. (2014), who analyzed SNPs associated with genes from three annotated pathways related to lactation. This most likely reflects the larger number of genes and SNPs included in the latter study.

The analysis suggests that the association with milk production traits contributed by the *JAK-STAT* pathway was significantly influenced by the inclusion of *SOCS* genes. The range of observed variation in the trait attributed to the optimized model for all *JAK-STAT* related SNPs was between 10 and 28%, and for *SOCS* genes alone was 6–24%. Biologically, this suggests that the *JAK-STAT*-*SOCS* genes play a significant role in determining performance in milk production and composition. The utility of *SOCS* gene-related SNPs in the model, may relate to the significant biological effect exerted by negative regulators of important functional pathways. The absence of *SOCS*2 in the model is likely to be explained by SNP allele frequencies, but could also reflect functional redundancy or cross talk in the *JAK-STAT* pathways.

To summarize this study, gene expression analyses supported an active role for members of the *SOCS* family of genes during the lactation cycle. SNPs linked to the *SOCS* and *JAK-STAT* pathway genes were useful in developing a model that accounted for significant variation in a number of important dairy traits. This study supports the view that there may be some merit in utilizing the SNPs around these genes for selection and genetic improvement schemes for dairy production traits.

## Conflict of Interest Statement

The authors declare that the research was conducted in the absence of any commercial or financial relationships that could be construed as a potential conflict of interest.

## Footnotes

1 http://adhis.com.au/

2 https://www.r-project.org/
